# Macroscopic dynamics of the ferroelectric smectic $A_F$ phase with $C_{\infty v}$ symmetry

**DOI:** 10.1140/epje/s10189-024-00406-x

**Published:** 2024-02-02

**Authors:** Helmut R. Brand, Harald Pleiner

**Affiliations:** 1 Department of Physics, University of Bayreuth, 95440 Bayreuth, Germany; 2 Max Planck Institute for Polymer Research, 55021 Mainz, Germany

## Abstract

**Abstract:**

We present the macroscopic dynamics of ferroelectric smectic *A*, smectic $A_F$, liquid crystals reported recently experimentally by three groups. In this fluid and orthogonal smectic phase, the macroscopic polarization, ${\textbf{P}}$, is parallel to the layer normal thus giving rise to $C_{\infty v}$ overall symmetry for this phase in the spatially homogeneous limit. A combination of linear irreversible thermodynamics and symmetry arguments is used to derive the resulting dynamic equations applicable at sufficiently low frequencies and sufficiently long wavelengths. Compared to non-polar smectic *A* phases, we find a static cross-coupling between compression of the layering and bending of the layers, which does not lead to elastic forces, but to elastic stresses. In addition, it turns out that a reversible cross-coupling between flow and the magnitude of the polarization modifies the velocities of both, first and second sound. At the same time, the relaxation of the polarization gives rise to dissipative effects for second sound at the same order of the wavevector as for the sound velocity. We also analyze reversible cross-coupling terms between elongational flow and electric fields as well as temperature and concentration gradients, which lend themselves to experimental detection. Apparently this type of terms has never been considered before for smectic phases. The question how the linear ${{\textbf{P}} \cdot \textbf{E}}$ coupling in the energy alters the macroscopic response behavior when compared to usual non-polar smectic *A* phases is also addressed.

**Graphical abstract:**

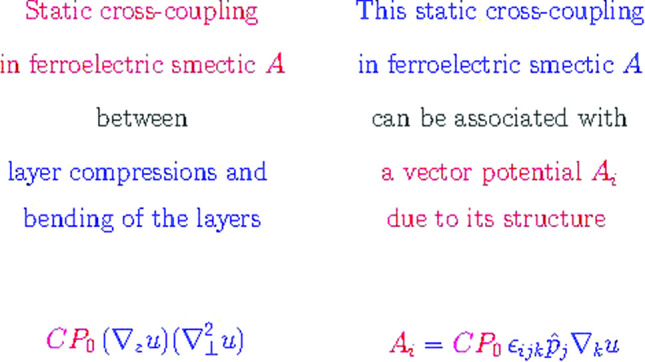

## Introduction

Last year the experimental observation of polar smectic *A* phases has been announced in Refs. [1–3]. In this phase, which is an orthogonal fluid smectic phase, the macroscopic polarization in the ground state is oriented parallel to the layer normal of the smectic planes and thus overall $C_{\infty v}$ symmetry prevails.

It is the purpose of the present paper to characterize the ferroelectric smectic *A* phase in the framework of macroscopic dynamics thus generalizing previous work on the macroscopic dynamics of usual non-polar smectic *A* phases [4–6] as well as of ferroelectric nematics [7–9].

To derive the macroscopic dynamic equations, we use the combination of irreversible thermodynamics and of symmetry arguments including the behavior of the macroscopic variables along with that of the associated currents and quasi-currents under time reversal, parity (spatial inversion) as well as under rigid rotations and Galilei transformations [5, 6, 10–12]. This approach has been applied to many condensed matter systems including spin waves in magnets [12, 13], nematic liquid crystals [4, 5, 12, 14], polymeric liquids [15–18] as well as superfluids including superfluid $^4$He [19, 20] and the superfluid phases of $^3$He [21–24].

The experimental discovery of ferroelectric smectic *A* phases followed about 5 years after ferroelectric nematic phases have been found experimentally [25, 26]. While ferroelectric (polar) nematic phases had been anticipated theoretically [7–9], this has apparently not been the case for ferroelectric smectic *A* phases. Over the last five years or so ferroelectric nematic phases have been further investigated in detail, both experimentally [27–40] as well as theoretically [41–43].

The paper is organized as follows. In Sect. 2, we discuss in detail our choice of the macroscopic variables, including the absolute value of the polarization, a concentration variable, and the electric charge density, in addition to standard variables of smectic A systems. The orientation of the polarization is expressed by gradients of the layer displacements. The static behavior follows from a total energy density expression in harmonic approximation that contains the static susceptibilities and allows for derivation of the conjugate fields. Section 3 is without electric fields, while Sect. 4 summarizes the electric field contributions. Compared to non-polar smectic A liquid crystals, there are cross-couplings among gradients of the polarization and the layer displacement on the one hand with changes of the density, entropy, concentration on the other. A very peculiar coupling of layer compression with layer bending is presented and its implications for elastic stresses are discussed in Sect. 8.1.

In Sect. 5, the dynamical equations are listed and the currents (in conservation laws) and quasi-currents (in balance equations) defined. Depending on their time reversal behavior, those currents are reversible or irreversible and explicitly shown in Sects. 6 and 7, respectively. Two examples from the reversible sector are further elucidated, first a coupling of the polarization with first and second sound in (Sect. 8.2) and, second, couplings of temperature or density gradients to reversible shear and extensional flow, characteristic for polar systems, in Sect. 8.3. In Sect. 9, we compare how some of the key features of this manuscript can be applied to other, related systems, like non-polar smectics, polar and non-polar nematics, when the order parameter is taken into account. We close with a summary and perspective in Sect. 10.

## Variables

In this section of the manuscript, we discuss the macroscopic variables, that must be taken into account for polar smectic *A* phases, also called smectic $A_F$, where $A_F$ refers to the ferroelectric nature of this phase. For certain polar aspects, we will make use of the previous work on the macroscopic description of polar nematics [7, 8]. Two fluid effects as they arise in immiscible mixtures [44, 45] are not the subject of this paper, since we will assume the presence of a miscible system.

Other variables are the entropy density, $\sigma$, the mass density, $\rho$, the density of linear momentum, $g_i$, and the macroscopic polarization, $P_i$. We split $P_i$ into its magnitude, *P*, and its direction, $\hat{p}_i$ with $P_i = P \hat{p}_i$, where $\hat{p}_i$ is a polar unit vector with $(\hat{p}_i)^2 = 1$. Rotations of the polarization are described by $\delta \hat{p}_i$. The strength of the polar order, *P*, is the order parameter, and $\delta P$ is not a conserved quantity. For very fast relaxation times, *P* does not couple to the macroscopic variables and will be no longer kept on the list of variables, while for sufficiently long relaxation times its dynamic equation will be incorporated.

Since the system we study, smectic $A_F$, has a layered structure, we keep *u*, the layer displacement, in our list of macroscopic variables, just as for non-polar smectic *A*. There is, however, an important difference: in the $A_F$ phase, $u = u_i \hat{p}_i$ is a component of the general displacement vector along the polar direction. Thus, *u* is scalar under inversion. In the non-polar case, $u_A = u_i \hat{n}_i$ involves the nematic direction and is therefore subject to the $\hat{n}_i$ $\rightarrow$ $-\hat{n}_i$ invariance characteristic for nematic order. In our description, $u_A$ is completely masked by *u*.

In this paper, we assume that the layer normal and the macroscopic polarization in equilibrium, $\hat{p}_i^0$, are parallel and fixed. As a result, rotations of the polarization are not independent from rotations of the layer and $\hat{p}_i$ is not an independent variable. In particular, in linear order

1 $$\begin{aligned} \delta \hat{ \varvec{p}} = - \varvec{\nabla }_\perp u \end{aligned}$$

with the transverse gradient vector $\varvec{\nabla }_\perp = \{\nabla _x, \nabla _y, 0 \}$ and the *z*-axis taken as the preferred direction. Of course, compression/dilation of the layers cannot be described by $\delta \hat{p}_i$.

The presence of impurities, contaminants, etc., can be taken care of by adding a concentration variable $\phi$. We assume the associated mass density, $\rho _s$, with $\rho _s = \rho \phi$ to be conserved, i.e., there are no chemical reactions involved [6].

The first law of thermodynamics relates changes of the variables to changes of the energy density $\varepsilon$ by the Gibbs relation [6, 10]

2 $$\begin{aligned} d\varepsilon&= T\, d\sigma + \mu \, d\rho + \varPi \, d\phi + h^u_i d \nabla _i u \nonumber \\&\quad + h^P dP + E_i d D_i + v_i dg_i \end{aligned}$$

The electric displacement field $D_i$ is related to the charge density $\rho _e$ by $\nabla _i D_i = - \delta \rho _e$ as the independent variable. The conjugate field $E_i$ is the external electric field. Our notation follows closely that of Refs. [8, 46].

The Gibbs relation contains the entropy density $\sigma$, representing the thermal degree of freedom, with its thermodynamic conjugate, the temperature *T*. Other conjugates are the chemical potential $\mu$, the osmotic pressure $\varPi$, the mean velocity $v_i = g_i / \rho$, the field $h^u_i$ is conjugate to $\nabla _i u$, and the molecular field $h^P$ associated with the magnitude of the polarization *P*.

## Statics-without fields

The static behavior of the macroscopic system studied here is conveniently described by the energy functional in harmonic approximation, Ref. [8, 47]. Disregarding electric fields for the moment and including the kinetic energy density, we get

3 $$\begin{aligned} \varepsilon&= + \frac{1}{2} c_P(\delta P)^2 + \frac{1}{2}K_{ij}^{(2)}(\nabla _i P)(\nabla _j P) \nonumber \\&\quad + \frac{1}{2} B (\nabla _z u)^2 + \frac{1}{2} K (\nabla _{\bot }^2 u)^2 + C P_0 (\nabla _z u ) (\nabla _{\bot }^2 u) \nonumber \\&\quad + (\nabla _z u)(d_1 \delta P + d_2 \nabla _z P) + (\nabla _\perp ^2 u) (d_3 \delta P + d_4 \nabla _z P) \nonumber \\&\quad + \frac{1}{2}c_{\rho \rho } (\delta \rho )^2 + \frac{1}{2}c_{\sigma \sigma }(\delta \sigma )^2 + \frac{1}{2}c_{\phi \phi }(\delta \phi )^2 \nonumber \\&\quad + c_{\rho \phi }(\delta \rho )(\delta \phi ) + c_{\rho \sigma }(\delta \rho )(\delta \sigma ) +c_{\sigma \phi }(\delta \sigma )(\delta \phi ) \nonumber \\&\quad + (\gamma _1 \delta \rho + \gamma _2 \delta \sigma + \gamma _3 \delta \phi ) \,\delta P \nonumber \\&\quad +(\theta _1 \delta \rho + \theta _2 \delta \sigma + \theta _3 \delta \phi ) \, \nabla _z P \nonumber \\&\quad + (\hat{\theta }_1 \delta \rho + \hat{\theta }_2 \delta \sigma + \hat{\theta }_3 \delta \phi ) \, \nabla _z u \nonumber \\&\quad + (\bar{\theta }_1 \delta \rho + \bar{\theta }_2 \delta \sigma + \bar{\theta }_3 \delta \phi ) \, (\nabla _{\bot }^2 u) + \frac{1}{2\rho } \varvec{g}^2 \end{aligned}$$

where $\nabla _z$ is a short hand notation for $\hat{p}_i^0 \nabla _i$. A $\delta$ denotes deviations from the equilibrium value, in particular $\delta P = P - P_0$, $\delta \hat{p}_i = \hat{p}_i - \hat{p}_i^0$, $\delta \phi = \phi - \phi _0$, $\delta \rho = \rho - \rho _0$ and $\delta T= T - T_0$. The Einstein summation convention is used throughout the paper.

Stability of the system requires a positive free energy. As a result, the static coefficients have to fulfil certain conditions; in particular for those related to the new variables $\delta P$ and $\nabla _i P$ we have $c_p > 0$, $K_{\perp }^{(2)} > 0$, $K_{||}^{(2)} > 0$ and $d_1^2 < c_p B$, $d_2^2 < K_{||}^{(2)} B$, $d_3^2 < c_p K$, $d_4^2 < K_{||}^{(2)} K$ and $\gamma _1^2 < c_p c_{\rho \rho }$, $\gamma _2^2 < c_p c_{\sigma \sigma }$, $\gamma _3^2 < c_p c_{\phi \phi }$ and $\theta _1^2 < K_{||}^{(2)} c_{\rho \rho }$, $\theta _2^2 < K_{||}^{(2)} c_{\sigma \sigma }$, $\theta _3^2 < K_{||}^{(2)} c_{\phi \phi }$.

Going beyond these inequalities in the estimate of static cross-coupling terms for room temperature complex fluids is a challenge. We note, however, that for uniaxial magnetic gels, Menzel [48] has presented a mesoscopic model of the cross-coupling between relative rotations and strains and evaluated the corresponding coupling parameter $D_2$ in term of the parameters of his mesoscopic model.

Even in harmonic approximation the energy density Eq. (3) can give rise to nonlinear effects, since material parameters generally are still functions of the state variables, like temperature, pressure, and polarization $P_0$.

The stiffness of order parameter variations is given by $c_P$. Inhomogeneous deviations of the polarization are described by energy contributions ($\sim K_{ij}^{(2)}$ which is of the standard uniaxial form) [6]. A surface term $\sim (\delta P)(\nabla _z P)$ has been suppressed.

Of the static contributions associated with layer displacements (gradients of *u*), the compression/dilation term $\sim B$ and the layer bending term $\sim K$ are of the standard form familiar from non-polar smectic *A* [4]. The contribution $\sim C P_0$ is specific for polar smectic *A* and vanishes in the non-polar case because of parity. This is made manifest by the factor $P_0$. It couples compression/dilation of the layers with bending of the layers. However, in linear order, the *C* term does not enter the Euler equation for the first variation of $\varepsilon$. There are four coupling terms between the polarization and layer displacement (coefficients $d_{1,2,3,4}$) unknown in non-polar smectics.

In addition, the energy density of a fluid binary mixture involving only $\delta \sigma$, $\delta \rho$ and $\delta \phi$ is as in the non-polar case. Specific for the $A_F$ phase are couplings between those variables and $\delta P$, $\nabla _z P$, $\nabla _z u$ and $\nabla _{\bot }^2 u$ with three new coefficients in each case. These coupling terms are absent in a smectic *A* phase, since they violate the ${\hat{\textbf{n}}} \rightarrow - {\hat{\textbf{n}}}$ invariance. The couplings with $\delta P$ have counter parts in solids that show piezoelectricity [49].

It is well-known that a phase with $\textrm{div} \,{\hat{\varvec{p}}} = \textrm{const}.$ (“splay phase”) does have a lower Ginzburg–Landau free energy (compared to the homogeneous state), but necessarily involves defects that increase the energy. The stability of such a splay phase depends, e.g., on boundary conditions and will not be considered here. For a hydrodynamic treatment of splay phases see [7]. Since we are dealing with a stable homogeneous equilibrium state here, the linear surface term, $\sim \textrm{div} \,{\hat{\varvec{p}}}$, can be neglected.

According to the Gibbs relation, Eq. (2) the conjugate quantities to the hydrodynamic and macroscopic variables follow from the free energy as variational or partial derivatives with respect to the appropriate variable, while all the other variables are kept constant. We provide a list of the conjugates in Sect. 4, with the field contributions included.

Naturally the harmonic approximation is a restriction in the sense that only sufficiently small deviations from the spatially homogeneous ground state are contained. Big changes such as, for example, a complete director reorientation as in the Frederiks transition [4], require a fully nonlinear analysis of all the variables involved.

Nonlinear generalizations of Eq. (3) contain the replacements $P_0 \rightarrow P$ and, particularly in the material tensors, $\hat{p}_i^0 \rightarrow \hat{p}_i$. The linear relation Eq. (1) is replaced by

4 $$\begin{aligned} \hat{ \varvec{p}} = \left( \nabla _x u, \nabla _y u, 1 - \frac{1}{2} (\varvec{\nabla }_\perp u)^2 \right) \end{aligned}$$

making sure that $\hat{p}_i^2 = 1$ up to quadratic order in gradients of *u*.

In addition, nonlinear rotations of the layers are accompanied by compression, which (in quadratic order) gives rise to the replacement

5 $$\begin{aligned} \nabla _z u \rightarrow \nabla _z u - \frac{1}{2} (\varvec{\nabla }_\perp u )^2 \end{aligned}$$

as is explained in Fig. 3b of Ref. [50]. This replacement applies in Eq. (3) to the terms $\sim B$ as well as $\sim C$, thus guaranteeing positivity of the energy, for $BK > C^2 P_0^2$.

## Statics-with external field

Applying a constant external electric field $E_i$ with magnitude *E*, the orientation of $\hat{p}_i^0$ in equilibrium will be parallel to the external field, $\hat{p}_i^0 = E_i / E$ due to the polarization electric coupling energy, $- {\varvec{P}\cdot \varvec{E}}$. For deviations from the preferred direction, a hydrodynamic polar orientation energy $(1/2) P_0 E (\delta \hat{p}_i)^2$ follows that describes the energetic penalty for not being in equilibrium. This latter expression follows from $\hat{p}_i \delta \hat{p}_i =0$ and $\hat{p}_i^0 \delta \hat{p}_i = -\frac{1}{2} (\delta \hat{p}_i)^2$. Finally, the polar orientation energy can be related to local layer rotations and is written as $(1/2) P_0 E \, (\varvec{\nabla }_\perp u)^2$ in harmonic approximation. Thus, the electric part of the orientational energy can be written

6 $$\begin{aligned} \varepsilon _{or} (E) = \frac{1}{2} K_E (\varvec{\nabla }_\perp u)^2 \end{aligned}$$

with $K_E = P_0 E - \epsilon _a E^2$. The non-polar dielectric orientation energy of the underlying smectic structure (with negative $\epsilon _a$) has been kept for completeness. In harmonic approximation, $K_E$ is constant.

Stability requires the necessary condition $\epsilon _a < P_0$, while $\epsilon _a <0$ is sufficient.

It is the linear field dependence on $P_0$ that can lead to a linear *E* dependence of certain material parameters. This is in contrast to ordinary nematics/smectics, where the material parameters can only be a function of $E^2$.

Ordinary non-polar nematics with a director field, $\varvec{\hat{n}}$, show flexoelectricity described by a contribution to the generalized energy of the form $e_1 (\delta _{ij}^{\bot } n_k - \delta _{jk}^{\bot } n_i) (\nabla _i n_j) E_k$ (for $\textrm{curl} \, \varvec{E}=0$) [4, 6]. For the polar $A_F$ phase, the flexoelectric energy reads $e_1 \hat{p}_i E_i (\textrm{div} \,{\hat{\varvec{p}}})$ and simply renormalizes the prefactor of the linear splay term, which we neglect in the free energy anyhow.

An applied constant magnetic field leads to an energy contribution $\frac{1}{2} \chi _a^m H^2 (\varvec{\nabla }_\perp u)^2$ involving the diamagnetic anisotropy $\chi _a^m$. It is quadratic in the field strength, since $H_i$ is odd under time reversal.

We add the electric orientational energy Eq. (6) to the field-free energy Eq. (3), $\tilde{\varepsilon }\equiv \varepsilon + \varepsilon _{or}$ and derive the conjugates by appropriate partial derivation. Writing $h^P = h^{'P} - \nabla _i\varPhi ^P_{i}$ and $h_i^u = h_i^{'u} - \nabla _j\varPhi ^u_{ij}$ we get

7 $$\begin{aligned} h^{'P}&= \frac{\partial \varepsilon }{\partial \delta P}\big \arrowvert _{\dots } = c_P \delta P + \gamma _1 \delta \rho + \gamma _2 \delta \sigma + \gamma _3 \delta \phi \nonumber \\&\quad + d_1 \nabla _z u + d_3 \nabla _\perp ^2 u \end{aligned}$$

8 $$\begin{aligned} \varPhi ^{P}_i&= \frac{\partial \varepsilon }{\partial (\nabla _i P)}\big \arrowvert _{\dots } = K_{ij}^{(2)} \nabla _j P + \hat{p}_i (d_2 \nabla _z u + d_4 \nabla _\perp ^2 u) \nonumber \\&\quad + \hat{p}_i (\theta _1 \delta \rho + \theta _2 \delta \sigma + \theta _3 \delta \phi ) \end{aligned}$$

9 $$\begin{aligned} h_i^{\prime u}&= \frac{\partial \tilde{\varepsilon }}{\partial \nabla _i u} \big \arrowvert _{\dots } = \hat{p}_i \bigl (B \nabla _z u + C P_0 \nabla _{\bot }^2 u + \hat{\theta }_1 \delta \rho \nonumber \\&\quad +\hat{\theta }_2 \delta \sigma +\hat{\theta }_3 \delta \phi + d_1 P + d_2 \nabla _z P \bigr )\quad \nonumber \\&\quad + \delta _{ij}^\perp K_E \nabla _j u \end{aligned}$$

10 $$\begin{aligned} \varPhi _{ij}^u&= \frac{\partial \varepsilon }{\partial \nabla _i \nabla _j u} \big \arrowvert _{\dots } = \delta _{ij}^{tr} ( K \nabla _{\bot }^2 u + C P_0 \nabla _z u + \bar{\theta }_1 \delta \rho \nonumber \\&\quad +\bar{\theta }_2 \delta \sigma +\bar{\theta }_3 \delta \phi + d_3 \delta P + d_4 \nabla _z P ) \end{aligned}$$

11 $$\begin{aligned} \delta \mu&= \frac{\partial \varepsilon }{\partial \delta \rho }\big \arrowvert _{\dots } = \gamma _1 \delta P + \theta _1 \nabla _z P + \bar{\theta }_1 \nabla _{\bot }^2 u + \hat{\theta }_1 \nabla _z u \nonumber \\&\quad + c_{\rho \rho }\delta \rho + c_{\rho \phi }\delta \phi + c_{\rho \sigma }\delta \sigma \end{aligned}$$

12 $$\begin{aligned} \delta T&= \frac{\partial \varepsilon }{\partial \delta \sigma }\big \arrowvert _{\dots } = \gamma _2 \delta P + \theta _2 \nabla _z P + \bar{\theta }_2 \nabla _{\bot }^2 u + \hat{\theta }_2 \nabla _z u \nonumber \\&\quad + c_{\sigma \sigma }\delta \sigma + c_{\rho \sigma }\delta \rho + c_{\sigma \phi }\delta \phi \end{aligned}$$

13 $$\begin{aligned} \delta \varPi&= \frac{\partial \varepsilon }{\partial \delta \phi }\big \arrowvert _{\dots } = \gamma _3 \delta P + \theta _3 \nabla _z P + \bar{\theta }_3 \nabla _{\bot }^2 u + \hat{\theta }_3 \nabla _z u \nonumber \\&\quad + c_{\phi \phi }\delta \phi + c_{\phi \rho }\delta \rho + c_{\phi \sigma }\delta \sigma \end{aligned}$$

14 $$\begin{aligned} v_i&= \frac{\partial \varepsilon }{\partial g_i} \big \arrowvert _{\dots } = g_i/\rho \end{aligned}$$

with $$\delta _{ij}^{tr} = \delta _{ij} - \hat{p}_i \hat{p}_j$$.

## Dynamics

In the following, we will disregard dynamical effects of the electric field, assuming $\varvec{\nabla } \times \varvec{E}=0.$ The dynamic equations have the form

15 $$\begin{aligned}&\dot{\sigma }+ \nabla _i (\sigma v_{i} + j_i^{\,\sigma {\mathrm R}} + j_i^{\,\sigma {\mathrm D}}) = \frac{2R}{T} \end{aligned}$$

16 $$\begin{aligned}&\dot{\rho }+ \nabla _i (\rho v_i) = 0, \end{aligned}$$

17 $$\begin{aligned}&\dot{g}_i + \nabla _j (g_i v_j + \psi \, \delta _{ij} - \hat{p}_i h_j^u + \sigma _{ij}^{\textrm{th}} + \sigma _{ij}^{\, \textrm{R}} + \sigma _{ij}^{\textrm{D}} ) = 0, \end{aligned}$$

18 $$\begin{aligned}&\dot{\phi }+ v_j \nabla _j \phi + \nabla _i ( j_i^{\phi \textrm{R}} + j_i^{\phi \textrm{D}}) = 0, \end{aligned}$$

19 $$\begin{aligned}&\dot{u} + v_j \nabla _j u -v_z+ X^{u \textrm{R}} + X^{u \textrm{D}} = 0, \end{aligned}$$

20 $$\begin{aligned}&\dot{P} + v_i \nabla _i P + X^{P \textrm{R}} + X^{P \textrm{D}} = 0, \end{aligned}$$

21 $$\begin{aligned}&\dot{\rho }_{e} + \nabla _j (\rho _{e} v_j) + \nabla _i ( j_i^{e \textrm{R}} + j_i^{e \textrm{D}}) = 0. \end{aligned}$$

The conserved quantities and the entropy density contain phenomenological currents ($\sim j_i$), while the quasi-currents ($\sim X$) are associated with spontaneously broken continuous symmetry variables or macroscopic variables. The superscripts *D* and *R* on the currents denote, respectively, the dissipative and reversible parts.

The energy conservation law

22 $$\begin{aligned} \dot{\varepsilon }+ \nabla _i (\varepsilon + \psi ) v_i + \nabla _i \bigl ( j_i^{\,\varepsilon {\textrm{R}}} + j_i^{\,\varepsilon {\textrm{D}}} \bigr ) = 0 , \end{aligned}$$

is redundant, since it follows from Eqs. (15)–(21) due to the Gibbs relation (2).

We use the pressure $\psi$ including the isotropic part of the Maxwell stress

23 $$\begin{aligned} \psi = \frac{\partial \,( \int \!\varepsilon dV )}{ \partial V}= -\varepsilon + \mu \rho + T\sigma + { \varvec{v}\cdot \varvec{g}} + D_i E_i \end{aligned}$$

and the off-diagonal terms of the Maxwell and the Ericksen-type stresses [51]

24 $$\begin{aligned} 2 \sigma ^{\textrm{th}}_{ij}&= - \left( E_iD_j + D_iE_j\right) + \varPhi _j^P \nabla _i P + \varPhi _i^P \nabla _j P \nonumber \\&\quad + \varPhi _{ki}^u \nabla _k \nabla _{j} u + \varPhi _{kj}^u \nabla _k \nabla _i u \end{aligned}$$

The Maxwell stress is of the standard form [11, 52] with $D_i = E_i + P_i$ and has been symmetrized with the help of the requirement that the energy density should be invariant under rigid rotations [6]. In detail, one first obtains directly from the condition of zero entropy production in Eq. (28)

25 $$\begin{aligned} \sigma ^{\textrm{th}}_{ij} = - D_j E_i + \varPhi _j^P \nabla _i P + \varPhi _{kj}^u \nabla _k \nabla _i u \end{aligned}$$

and uses the requirement of rotational invariance of the Gibbs relation [5]. Compare also Ref. [5] for a detailed exposition.

With a redefinition of the pressure, $\psi = \tilde{\psi }+ (1/2)E^2$, another useful form of the momentum conservation Eq. (17) can be found

26 $$\begin{aligned}&\dot{g}_i + \nabla _j \bigl (g_i v_j + \tilde{\psi }\, \delta _{ij} - \hat{p}_i h_j^u + \varPhi _j^P \nabla _i P + \sigma _{ij}^{\, \textrm{R}} + \sigma _{ij}^{\,\textrm{D}} \bigr ) \nonumber \\&\quad =\rho _e E_i + P_j \nabla _j E_i, \end{aligned}$$

with the Coulomb and Kelvin external forces as source terms. Using the Gibbs relation Eq. (2), the pressure gradient can be written as

27 $$\begin{aligned} \nabla _i \tilde{\psi }= \rho \nabla _i \mu +\sigma \nabla _i T - \varPi \nabla _i \phi + P_j \nabla _i E_j \end{aligned}$$

The source term of Eq. (15) contains *R*, the dissipation function, which represents the energy dissipation of the system. Due to the second law of thermodynamics, *R* must satisfy $R\ge 0$: For reversible processes, this dissipation function is equal to zero, while for irreversible processes it must be positive

28 $$\begin{aligned} 2 R&= -j_i^{\sigma *} \nabla _i T - j_{i}^{\phi *} \nabla _{i} \varPi - \sigma _{ij}^{*} \nabla _j v_i - j_i^{e*} E_i \nonumber \\&\quad - X^{u *} \nabla _i h_i^u + X^{P*} h^P \ge 0 \end{aligned}$$

where positive entropy production ($R >0$) applies for $* = \textrm{D}$ and vanishing entropy production ($R = 0$) for $*=\textrm{R}$. The superscripts $\textrm{D}$ refer to the dissipative part of the currents and quasi-currents in Eqs. (15)–(21) while the superscripts $\textrm{R}$ refer to the reversible parts in Eqs. (15)–(21).

The phenomenological currents and quasi-currents are the sum of the reversible and the dissipative part, as can be seen in Eqs. (15)–(21). The various transport contributions in Eqs. (15)–(21) (as well as $\psi$ and $\sigma ^{\textrm{th}}_{ij}$) are reversible and add up to zero in the entropy production.

These phenomenological currents and quasi-currents are treated in the following subsections within ‘linear irreversible thermodynamics’ (guaranteeing general Onsager relations), i.e., as linear relations between currents and thermodynamic forces. The resulting expressions are nevertheless nonlinear, since all material parameters can be functions of the scalar state variables (e.g., $\sigma$, $\rho$, *P*, $\phi$).

The form of Eq. (18) reflects the assumption that the impurity density is conserved, while Eq. (21) describes the polar order parameter modulus as a slowly relaxing quantity (similar to, e.g., the nematic order parameter modulus [53] or the superfluid degree of order [20, 54]).

## Reversible currents

To obtain the reversible currents, one expands all currents and quasi-currents systematically into the thermodynamic forces/conjugates taking into account the behavior under time reversal, spatial inversion, rigid rotations and, most importantly, zero entropy production. For a more detailed exposition of the method, we refer to Ref. [6]. For the reversible dynamic behavior of our macroscopic system, we obtain the following expressions for the reversible currents containing phenomenological parameters

29 $$\begin{aligned} j_i^{\,\sigma \textrm{R}}&= \varphi _{ijk}^{\sigma } A_{jk}, \end{aligned}$$

30 $$\begin{aligned} j_i^{\,e \textrm{R}}&= \varphi _{ijk}^{e} A_{jk}, \end{aligned}$$

31 $$\begin{aligned} \sigma _{ij}^{\, \textrm{R}}&= \lambda _{ij}^P h^P - \varphi _{kji}^\sigma \nabla _k T - \varphi _{kji}^\phi \nabla _k \varPi - \varphi _{kji}^{e} E_k, \end{aligned}$$

32 $$\begin{aligned} X^{P \textrm{R}}&= \lambda _{ij}^P A_{ij}, \end{aligned}$$

33 $$\begin{aligned} X^{u \textrm{R}}&= 0, \end{aligned}$$

34 $$\begin{aligned} j_i^{\phi \textrm{R}}&= \varphi _{ijk}^{\phi } A_{jk}, \end{aligned}$$

with the symmetrized velocity gradient $A_{ij}$, where $2A_{ij} = \nabla _i v_j + \nabla _j v_i$. There is a coupling of the modulus of the polarization and the density of linear momentum provided by the tensor

35 $$\begin{aligned} \lambda _{ij}^P = \lambda ^P_1 \delta _{ij}^{\bot } + \lambda ^P_2 \hat{p}_i \hat{p}_j \end{aligned}$$

The experimental implications of this coupling are discussed in Sect. 8.2.

A second class of reversible couplings are provided by the third-rank material tensors $\varphi _{ijk}^{\alpha }$ for $\alpha \in \{\sigma , e, \phi \}$ with the structure

36 $$\begin{aligned} \varphi _{ijk}^{\alpha } = \varphi ^{\alpha }_1 \hat{p}_i \hat{p}_j \hat{p}_k + \varphi ^{\alpha }_2 \hat{p}_i \delta _{jk}^{\bot } + \varphi ^{\alpha }_3 (\hat{p}_j \delta _{ik}^{\bot } + \hat{p}_k \delta _{ij}^{\bot }) \end{aligned}$$

They connect currents of scalar conserved quantities with $A_{ij}$ and, reciprocally, their conjugate forces with the stress tensor $\sigma _{ij}$. Such couplings require the presence of a parity breaking vector (compare, for example, Ref. [9]). All parts of the tensors $\varphi _{ijk}^{\alpha }$ therefore contain an odd number of $\hat{p}_i$ in order to restore parity symmetry. These reversible dynamic cross-coupling terms are absent in non-polar systems such as non-polar smectic *A*. In Sect. 8.3, we discuss consequences of those contributions that can be detected experimentally.

## Dissipative currents

To describe dissipative processes, it is convenient to expand the dissipation function, *R*, the source term in the dynamic equation for the entropy density, Eq. (15), into a positive definite expression quadratic in the thermodynamic forces. Then taking variational derivatives (or partial derivatives when applicable) of *R* with respect to forces, one obtains linear relations (listed below) between the currents and the quasi-currents on the one hand and thermodynamic forces on the other. The entropy production is a scalar under all transformations compatible with symmetry including time reversal, spatial parity, and rigid rotations. For a detailed exposition of the method, we refer to Ref. [6]. The dissipative dynamic behavior of our macroscopic system is characterized by the dissipation function *R*

37 $$\begin{aligned} 2 R&= \kappa _{ij} (\nabla _i T) (\nabla _j T) + D_{ij} (\nabla _i \varPi )(\nabla _j \varPi ) \nonumber \\&\quad + 2D^{T \varPi }_{ij} (\nabla _i T)(\nabla _j \varPi ) + \sigma _{ij}^E E_i E_j \nonumber \\&\quad + 2D^{T E}_{ij} (\nabla _i T) E_j + 2 D_{ij}^{\varPi E} (\nabla _i \varPi ) E_j \nonumber \\&\quad + \frac{1}{\gamma _u} (\nabla _i h_i^u)(\nabla _j h_j^u) + \nu _{ijkl} (\nabla _j v_i)(\nabla _l v_k) \nonumber \\&\quad + 2 D^{PE} E_z h^{P} + 2 D^{u\varPi } (\nabla _z \varPi ) (\nabla _i h_i^u) + b_{||} h^{P} h^{P} \nonumber \\&\quad + 2 D^{PT} ( \nabla _z T) h^{P} + 2D^{P\varPi }( \nabla _z\varPi )h^{P} \nonumber \\&\quad + 2 D^{uT} (\nabla _z T) (\nabla _i h_i^u) + 2 D^{uE} E_z (\nabla _i h_i^u) \end{aligned}$$

where $E_z$ is a short-hand notation for $\hat{p}_i E_i$. The tensors $\kappa _{ij}$, $D^{T\varPi }_{ij}$, $D_{ij}$, $\sigma _{ij}^E$, $D_{ij}^{TE}$ and $D_{ij}^{\varPi E}$ as well as $\nu _{ijkl}$ are of the standard uniaxial form for second and fourth ranks tensors [6, 49]. The contribution $\sim b_{||}$ in the entropy production describes the relaxation of the polarization modulus *P*. This term has an analog in ordinary nematics (with the order parameter modulus included). Specific for polar systems including polar nematics, polar nematics with a solvent and polar smectic $A_F$ are the dissipative cross-couplings between polarization on the one hand and gradients of temperature, gradients of osmotic pressure and electric fields on the other governed by the material parameters $D^{PT}$, $D^{P\varPi }$ as well as $D^{PE}$. These contributions can only exist in polar system, since they are odd in $\hat{p}_i$.

The positivity of *R* requires certain conditions on the dissipative parameters, in particular, $b_{||} > 0$ and $(D^{PE})^2 < \sigma _{||}^E b_{||}$, $(D^{PT})^2 < \kappa _{||} b_{||}$, and $(D^{P\varPi })^2 < D_{||} b_{||}$. To go in general beyond these positivity requirements is highly nontrivial. In fact, we are not aware of any systematic approach to evaluate transport coefficients quantitatively for room temperature fluid condensed systems in the bulk. We would like to mention, however, that in superfluid $^3$He, which arises at temperatures below 3 mK in the bulk, one can evaluate transport coefficients quantitatively [55], since one has a small expansion parameter, namely $\varDelta /\varepsilon _F \sim 10^{-3}$, where $\varDelta$ is the energy gap and where $\varepsilon _F$ is the Fermi energy.

To obtain the dissipative parts of the currents and quasi-currents, we take the partial derivatives of *R* with respect to the appropriate thermodynamic force

38 $$\begin{aligned} j^{\sigma \textrm{D}}_i = - \frac{\partial R}{\partial (\nabla _i T)}\big \arrowvert _{\dots }&= - \kappa _{ij} \nabla _j T - D^{\varPi E}_{ij} \nabla _j\varPi \nonumber \\&\quad - D_{ij}^{T E} E_j - D^{TP} \hat{p}_i h^P \nonumber \\ {}&\quad - D^{uT} \hat{p}_i (\nabla _j h_j^u) \end{aligned}$$

39 $$\begin{aligned} j^{e \textrm{D}}_i = - \frac{\partial R}{\partial E_i }\big \arrowvert _{\dots }&= - \sigma _{ij}^E E_j - D^{\varPi E}_{ij} \nabla _j\varPi \nonumber \\ {}&\quad - D_{ij}^{T E} \nabla _j T - D^{PE} \hat{p}_i h^P \nonumber \\ {}&\quad - D^{uE} \hat{p}_i (\nabla _j h_j^u) \end{aligned}$$

40 $$\begin{aligned} j^{\phi \textrm{D}}_i = - \frac{\partial R}{\partial (\nabla _j\varPi )}\big \arrowvert _{\dots }&= - D_{ij} \nabla _j\varPi - D^{T\varPi }_{ij} \nabla _j T \nonumber \\&\quad - D_{ij}^{\varPi E} E_j - D^{P\varPi } \hat{p}_i h^P \nonumber \\&\quad - D^{u\varPi } \hat{p}_i (\nabla _j h_j^u) \end{aligned}$$

41 $$\begin{aligned} \sigma ^{\textrm{D}}_{ij} = - \frac{\partial R}{\partial (\nabla _jv_i)}\big \arrowvert _{\dots }&= - \nu _{ijkl}A_{kl} \end{aligned}$$

42 $$\begin{aligned} X^{P \textrm{D}} = \frac{\partial R}{\partial h^P}\big \arrowvert _{\dots }&= b_{||} h^P + D^{P\varPi } \nabla _z \varPi \nonumber \\ {}&\quad + D^{PE} E_z + D^{PT} \nabla _z T \end{aligned}$$

43 $$\begin{aligned} X^{u \textrm{D}} = - \frac{\partial R}{\partial (\nabla _i h_i^u) }\big \arrowvert _{\dots }&= - \frac{1}{\gamma _u} \nabla _i h_i^u - D^{u\varPi } \nabla _z \varPi \nonumber \\ {}&\quad - D^{uE} E_z - D^{uT} \nabla _z T \end{aligned}$$

## Possible static and dynamic experiments

### On the unusual static coupling between compression and deformations of the layering

As pointed out briefly in Sect. 3, the most interesting new static cross-coupling term when compared to usual smectic *A* is the contribution $C P_0 (\nabla _z u ) (\nabla _{\bot }^2 u)$ in Eq. (3), which couples layer compressions to a bending of the layers.

From Eqs. (9) and (10), it is easy to see that this novel contribution does not generate bulk effects in the dissipative currents, Eqs. (38)–(43), since its first variational derivative, $\nabla _i h_i^u$ vanishes identically. On the other hand, the *C* term is not a pure surface term, in contrast, e.g., to $\nabla _i P_i$ in the energy. The nonlinear, rotationally invariant generalization of $\nabla _z u$ in the *C* term using Eq. (5), leads to the same conclusions. We are not aware of any other cross-coupling term in the hydrostatic regime for which such a situation arises.

The only instance where $h_i^u$ appears (and not its divergence) is the stress tensor $\sigma _{ij}$ in Eq. (17), where $\hat{p}_i h_j^u$ represents the elastic tensions. It consists of two parts,

44 $$\begin{aligned} \sigma _{ij}^C = C P_0 \,\, (\hat{p}_i \hat{p}_j \nabla _{\bot }^2 u - \hat{p}_i \delta _{jk}^{tr} \nabla _k \nabla _z u) \end{aligned}$$

describing a relative compression—shear tension weighted with $\nabla _{\bot }^2 u$ (for the part of compressional tensions) and $\nabla _\perp \nabla _z u$ (for the contribution from shear tensions). Clearly, the challenge lies in an experimental detection of these two effects.

To come closer to a physical interpretation of Eq. (44) or generally of the contribution $\sim C$, we can introduce a vector potential $A_i$ by $h_i^u = \epsilon _{ijk} \nabla _j A_k$ similar to the condition of incompressibility in hydrodynamic flow or to the Coulomb gauge in electrodynamics. We find

45 $$\begin{aligned} A_i = C P_0 \epsilon _{ijk} \hat{p}_j \nabla _k u \end{aligned}$$

which looks rather simple.

### Sound-like excitations

In simple fluids, ordinary sound is known as the only propagating low-*k* excitation, $\exp { i(k_i r_i - \omega t)}$, with $\omega ^2 \sim k^2$. It is related to momentum conservation, isotropic, and results from reversible couplings of $\nabla _i v_i$ with $\delta \rho$ and $\delta \sigma$ via the isotropic pressure in Eq. (17).

In a smectic A liquid crystal, polar or non-polar, there is an additional propagating mode, sometimes called second sound. It is due to the spontaneously broken translational symmetry along the normal of the layers (the *z* direction). This one-dimensional compression/dilation mode, with susceptibility *B*, Eq. (3), is anisotropic and leads to an anisotropic part in the first sound spectrum. It involves excitations of $\nabla _i v_i$ and $\nabla _z u$.

In a polar smectic phase, $A_F$, the polarization $\delta P$ provides additionally a low-*k* coupling with $\nabla _i v_i$ and $\nabla _z v_z$ via $\lambda _{ij}^P$ in Eqs. (31) and (32). As a result, both sound-like excitations are more complicated.

Disregarding dissipation for the moment, the solvability condition for the linearized equations of motion, Eqs. (15)–(20) leads after some trivial algebra to the dispersion relations for first sound

46 $$\begin{aligned} \omega _1^2 = c_{10}^2 k^2+ \frac{B}{\rho _0} \frac{k_z^4}{k^2} +\frac{c_P}{\rho _0} \frac{(\lambda _1^P k^2 + \lambda _a^P k_z^2)^2}{k^2} \end{aligned}$$

with $\lambda _a^P = \lambda _2^P - \lambda _1^P$ and $k_z$ short-hand for $\hat{p}_i k_i$. For the isotropic first sound velocity (of simple fluids), we get $\rho _0 c_{10}^2 = c_{\rho \rho }^2 \rho _0^2 + 2 c_{\rho \sigma }^2 \rho _0 \sigma _0 + c_{\sigma \sigma }^2 \sigma _0^2$ in our representation.

The second contribution ($\sim B$) shows a uniaxial dependence on the angle $\vartheta$ between $k_i$ and $\hat{p}_i$, $k^2 \cos ^4 \vartheta$. The last contribution ($\sim c_P$) reflects the anisotropy of the material tensor $\lambda _{ij}^P$. In case of vanishing anisotropy in the $\lambda _{ij}^P$ tensor, $\lambda _a^P =0$, it adds to the isotropic part of first sound velocity, $c_{10}^2 \rightarrow c_{10}^2 + c_P (\lambda _1^P)^2$, while for $\lambda _1^P = 0$ the compression modulus is effectively renormalized $B \rightarrow B + c_P (\lambda _a^P)^2$. In the general case, first sound anisotropy has contributions $\sim k^2 \cos ^4 \vartheta$ and $\sim k^2 \cos ^2 \vartheta$.

The rather simple form of Eq. (46) is due to our assumption that $c_{10}^2 k^2$ is the dominant contribution to first sound. In addition, we have neglected $\gamma _{1,2,3}$, since those couplings only lead to isotropic contributions.

For second sound, we find two parts

47 $$\begin{aligned} \omega _{20}^2 = (c_{B}^2 + c_{2\lambda }^2 ) \frac{k_\perp ^2 k_z^2 }{k^2} \equiv c_2^2 k^2 \sin ^2 \vartheta \cos ^2 \vartheta \end{aligned}$$

with $c_{B}^2 = B/\rho$, related to the smectic compression mode, and $c_{2\lambda }^2 = (c_P/\rho ) (\lambda _a^P)^2$, related to the $\lambda _{ij}^P$ coupling. The perpendicular wave vector is $k_\perp ^2 = k^2 \sin ^2 \vartheta$.

Second sound does not have an isotropic part and is a manifestation of the couplings among $\delta P$, $\nabla _i v_i$, $\nabla _z v_z$ and $\delta \psi$. This mode vanishes for $k_i$ that is either parallel or perpendicular to $\hat{p}_i$.

Concerning dissipation, it is well known that there is no damping for ordinary first sound and the smectic compressional wave in lowest order of the wave vector $\omega \sim k$ and is therefore often neglected. This is different for the coupling of the polarization $\sim \lambda _{ij}^P$, since the relaxation of $\delta P$, given by $\dot{P} + \lambda _{ij}^P A_{ij} + b_{||} c_P P =0$ (in lowest order in *k*) enters the dispersion relations discussed above.

For second sound, we find the implicit relation

48 $$\begin{aligned} \omega _{2}^2 = \left( c_{B}^2 + \frac{\omega _2}{\omega _2 + i b_{||} c_P} c_{2\lambda }^2 \right) \frac{k_\perp ^2 k_z^2 }{k^2} \end{aligned}$$

where the imaginary part ($\sim b_{||}$) indicates damping. As expected, only the $\lambda _{ij}^P$ contribution is damped.

Approximate solutions for weak and strong damping, respectively, are obtained by replacing in Eq. (48) the factor

49 $$\begin{aligned} \begin{array}{lll} \left( \frac{\omega _2}{\omega _2 + i b_{||} c_P} c_{2\lambda }^2 \right) &{}\quad \textrm{by} &{}\quad c_{2\lambda }^2 \left( 1 - i \, \frac{b_{||} c_P}{\omega _{20}} \right) \\ &{}&{}\quad \textrm{if} \quad \omega _{20}^2 \gg b_{||}^2 c_P^2 \end{array} \end{aligned}$$

and

50 $$\begin{aligned} \begin{array}{lll} \left( \frac{\omega _2}{\omega _2 + i b_{||} c_P} c_{2\lambda }^2 \right) &{}\quad \textrm{by} &{}\quad c_{2\lambda }^2 \, \frac{\omega _{20}}{b_{||} c_P} \left( -i + \frac{\omega _{20}}{b_{||} c_P} \right) \\ &{}&{}\quad \textrm{if} \quad \omega _{20}^2 \ll b_{||}^2 c_P^2 \end{array} \end{aligned}$$

In the case of small $b_{||}$, the real (propagating) part is unchanged, $\mathfrak {Re} (\omega _2^2) = \omega _{20}^2$, and the imaginary (relaxing) part is basically the polarization relaxation $\mathfrak {Im} (\omega _{2}^2) \sim c_P b_{||}$. For large $b_{||}$, the real part of $\omega _2^2$ is dominated by $c_B^2$, while the imaginary part is due to $c_{2\lambda }^2$. For very large $b_{||}$, second sound becomes real and is the ordinary smectic compression mode ($\omega _2^2 \sim c_B^2$).

The influence of the relaxation of $\delta P$ in the first sound spectrum shows up only in the part ($\sim c_P$) of Eq. (46). The results for second sound discussed above can be taken over accordingly for this part of first sound.

### Reversible shear and elongational flows

Here, we discuss some of the implications of the reversible cross-coupling terms described by the tensors $\varphi _{ijk}^{\alpha }$, Eq. (36), between velocity gradients and gradients of temperature ($\alpha = \sigma$) and concentration ($\alpha =\phi$), or electric fields ($\alpha = e$). We take $\hat{p}_i$ parallel to the *z*-direction with the perpendicular layering in the $x - y$ planes resembling a free-standing film geometry.

Among the external flow patterns that lead to those couplings in an $A_F$ phase, there are basically two configurations. First, a simple shear flow (with shear rate *S*) in a plane containing the preferred direction *z* and an orthogonal direction, *y*, meaning $v_i^{(1)} = S y \,\delta _{iz}$ or $2 A_{jk} = S ( \delta _{jy} \delta _{kz} + \delta _{jz} \delta _{ky})$, Eq. (29) leads to a heat current of the form

51 $$\begin{aligned} j_y^{\sigma ,\textrm{R}} = \varphi _3^{\sigma } S \end{aligned}$$

and vanishing components $j_z^{\sigma ,R}$ and $j_x^{\sigma ,R}$. Therefore, the heat current is within the smectic layering and along the applied velocity. Of course, the *y*-direction can be any in-layer direction.

The second example is uniaxial elongational or compressional flow in a in-layer direction, $v_i^{(2)} = L y \,\delta _{iy}$ or $A_{jk} = L \delta _{jy} \delta _{ky}$ that results in a heat current with the single component

52 $$\begin{aligned} j_z^{\sigma ,\textrm{R}} = \varphi _2^{\sigma } L \end{aligned}$$

across the layers along the preferred direction.

In principle, also another uniaxial elongational flow (along the preferred direction), $v_i^{(3)} = L^\prime z \,\delta _{iz}$ or $A_{jk} = L^\prime \delta _{jz} \delta _{kz}$ results in a heat current $j_z^{\sigma ,R} = \varphi _1^{\sigma } L^\prime$ across the layers. However, this flow is incompatible with a constant layer spacing and would be challenging in measuring $\varphi _1^\sigma$. Perhaps one can achieve this goal by applying an oscillatory flow of small amplitude.

Analogously, we obtain concentration and electric currents of the same form (replacing in the $\varphi$ tensor superscript $\sigma$ by $\phi$ and *e*, respectively,

Reciprocally one can apply a temperature, a concentration gradient or an electrical field to a sample and then obtain non-vanishing elements of the stress tensor. For a temperature gradient parallel to ${\hat{\textbf{p}}}$, $\nabla _z T = G_{\parallel }$, we find

53 $$\begin{aligned} \sigma _{zz}&= \varphi _1^{\sigma } G_{\parallel } \end{aligned}$$

54 $$\begin{aligned} \sigma _{xx} = \sigma _{yy}&= \varphi _2^{\sigma } G_{\parallel } \end{aligned}$$

55 $$\begin{aligned} \sigma _{xz} = \sigma _{xy} = \sigma _{yz}&\equiv 0 \end{aligned}$$

For a temperature gradient perpendicular to ${\hat{\textbf{p}}}$, $\nabla _x T = G_{\perp }$, we find

56 $$\begin{aligned} \sigma _{xz} = \sigma _{zx}&= \varphi _3^{\sigma } G_{\perp } \end{aligned}$$

57 $$\begin{aligned} \sigma _{xx} = \sigma _{yy} = \sigma _{zz} = \sigma _{xy}&\equiv 0 \end{aligned}$$

Such stresses can lead to flows via the dynamic equation for the density of linear momentum only for spatially inhomogeneous external temperature or concentration gradients or inhomogeneous electric fields.

## Comparison with other liquid crystal systems

In the smectic $A_F$ phase, we have found a novel static cross-coupling between compression of the layering and bending of the layers, Sect. 8.1. A similar coupling can be expected for the hypothetical ferroelectric analog of the tilted smectic *C* phase. In addition, a hypothetical ferroelectric $C_M$ phase, which can be viewed as a smectic $A_F$ phase that has an additional preferred (nematic or polar) direction within the layers, should allow for a *C*-type coupling.

To the best of our knowledge, there is no other liquid crystalline system to date that allows for the *C* term. It does not lead to bulk forces, since its first variational derivative vanishes, but it is not a simple boundary term. It gives a rather complicated contribution to the stress tensor, Sect. 8.1.

Other effects, discussed above, have counterparts in various liquid crystal systems. The modification of the sound mode spectrum due to a reversible coupling between flow and the polarization (the polar order parameter), Sect. 8.2, is present analogously in non-polar smectics, if there, the nematic order parameter (usually called *S*) relaxes slowly and is taken into account as a macroscopic variable. In the standard description of non-polar smectic *A* (and discotic/columnar) phases [56], such sound modifications are not contained. Similarly, in polar and non-polar nematics with slowly relaxing polar or nematic order, respectively, the reversible coupling via $\lambda _{ij}^P$ in Ref. [8] or $\beta _{ij}$ in Ref. [57] between flow and the appropriate order parameter exists. As a consequence first sound becomes anisotropic and second sound arises as a propagating mode (with slowly relaxing amplitude). They are easier measurable (than in smectic systems), since there are no layer compression modes in nematics.

The reversible couplings of temperature or density gradients to reversible shear and extensional flow, Sect. 8.3, are characteristic for systems with polar order or tetrahedral (octupolar) order. The former case includes polar nematics, Ref. [8], the latter comprises tetrahedral phases, like the $T_d$, $D_{2d}$, $S_4$ and $D_2$ phase, Ref. [58]. The tetrahedral order parameter is a rank 3 tensor without inversion symmetry.

## Summary and perspective

In this work, we have focused on the macroscopic dynamics of ferroelectric smectic *A*, smectic $A_F$, liquid crystals reported recently experimentally. In this fluid and orthogonal smectic phase, the macroscopic polarization, ${\textbf{P}}$, is parallel to the layer normal thus giving rise to $C_{\infty v}$ overall symmetry for this phase in the spatially homogeneous limit. A combination of linear irreversible thermodynamics and symmetry arguments has been used to derive the resulting dynamic equations applicable at sufficiently low frequencies and sufficiently long wavelengths.

There are several directions into which one can generalize the analysis presented in this paper. First of all one could produce a material composed of chiral molecules, which will most likely lead to a ferroelectric A phase, which will also break mirror symmetry in all three spatial dimensions, in contrast to the smectic $A_F$ phase analyzed here—one could call this phase smectic $A_F^*$.

In a step toward soft solids and gels, it would be natural to study the influence of a network leading to ferroelectric smectic *A* elastomers and gels. Such a material would combine the material and mechanical properties of smectic *A* elastomers studied in the past by Nishikawa et al. [59, 60] with ferroelectricity along the layer normal of the smectic layering. These systems would be very interesting, because it has been shown in Refs. [59, 60] for usual smectic *A* elastomers that the in-plane elasticity is about two orders of magnitude smaller than the elastic modulus for compression due to the layered structure.

Another direction to go into would be the investigation of two fluid effects in smectic liquid crystals, both for non-polar usual smectic *A* phases as well as for ferroelectric smectic $A_F$ in a solvent each. Even for polar nematics, such a study is rather recent [43]. In such systems, relative motions between the layering and the solvent will be of interest, in particular for lyotropic systems, which can have a large layer spacing compared to a molecular length and even becoming comparable to optical wavelengths. This issue has not been addressed in the literature so far.

We close this perspective by mentioning that the investigation of ferromagnetic instead of ferroelectric smectic *A* phases would open a new field of study altogether, since new dynamic cross-coupling terms, reversible as well as irreversible ones, would become possible in such a material, since ferroelectric and ferromagnetic systems differ re. electric polarization, ${\textbf{P}}$, and spontaneous magnetization, ${\textbf{M}}$, by their different behavior under both, parity and time reversal. Experimental studies on ferrosmectics are rather rare (compare in particular [61, 62]) and are so far confined to systems that are not ferromagnetic, since the particles used were neither plate- nor rod-like. This situation is in strong contrast to experimental and theoretical studies of ferronematics and ferromagnetic nematics, a field that has flourished over the last decade [63–74].

## Data Availability

Data sharing not applicable to this article.
